# Children’s complex health: Maternal experiences of care and decision making

**DOI:** 10.1177/13674935231158456

**Published:** 2023-03-01

**Authors:** Eleanor Willis, Rosemary Godbold

**Affiliations:** 1 7616 East and North Hertfordshire NHS Trust, Stevenage, Hertfordshire, UK; 2 3769 University of Hertfordshire, Hatfield, UK

**Keywords:** Community health nursing, long-term care, decision making, mothers, qualitative research

## Abstract

An increasing number of children with complex life-limiting and life-threatening conditions are being cared for at home by their parents. Negative impacts on maternal health are now being recognised. This study sought to voice parental experiences to determine what matters most; explore day-to-day decision making and Advance Care Planning; and to inform local service development. Seven mothers from one community nursing service were interviewed using a semi-structured qualitative approach during the Coronavirus disease 2019 pandemic. Seven data-driven themes were identified following six phase thematic analysis: cherishing normality; navigating the system; being proactive; meaningful connections; beginner to expert – and back; they’re not any child – they’re my child; and Coronavirus disease 2019 pandemic. Practice implications include early discussion of what matters most and benefits of written plans to ensure fair access to treatment for children with complex health. Mothers highlighted that sharing their story enhanced their sense of coping and purpose. Increased support at times of vulnerability and permission to explore decisions were highly valued.

## Introduction

More children than ever before are living with increasingly complex health needs at home (Pinney, 2017). Parents are expected to routinely undertake clinical tasks, maintain communication with multiple teams and negotiate challenging systems of care (Carter and Bray, 2017). Parents assume many additional roles in order to care for their children at home, balancing clinical expertise with parenting (Mafuba, 2015). This patient population is often considered invisible, with daily challenges unseen by acute services (McCann, 2015).

Detrimental effects on maternal physical and mental health are evident, including increased risk of depression, cardiac disease and reduced life expectancy (Fraser et al., 2020). However, health and social service professionals have not investigated what measures are needed to address these issues which have been exacerbated by the Coronavirus disease 2019 (COVID-19) (Hartley et al., 2021; Gallegos et al., 2022). Exploration of patient and carer needs is required to underpin design and delivery of appropriate, high quality services (Kings Fund, 2010).

**Figure 1.**
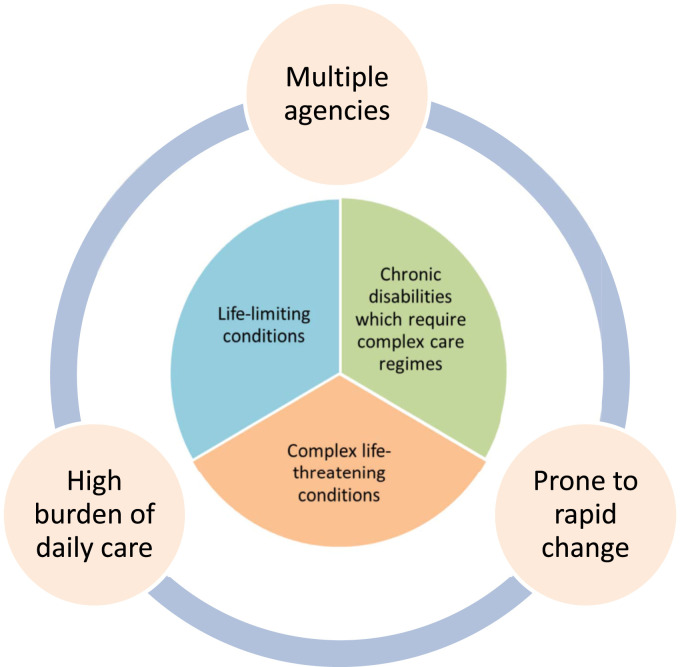
Cohort definition of children’s complex health.

No single definition of children’s complex health exists and various definitions are used by health and care services (Brenner et al., 2018). Yet what unites them is acknowledgement that this group of children have exceptional health needs (Brenner et al., 2021; NICE, 2022). Children with a life-limiting or life-threatening condition do not all have complex needs; however, this assumption is not well documented (Brenner et al., 2018). The number of agencies involved in caring and supporting such children is clearly evident (Carter and Bray, 2017; Coad et al., 2015) and burdens of daily care needs carried out by parents have been shown to directly impact their experience (Fairfax et al., 2019; Woodgate et al., 2015). Children with complex health do not fit generic pathways of care and some receive innovative treatment making it difficult to anticipate their patient journey (Carter, 2016). Therefore for the purpose of this study, a cohort definition was developed to inform recruitment and promote transferability of findings (Figure 1).

The cohort definition identifies complex health as encompassing life-limiting conditions, chronic disabilities requiring complex regimes to maintain stability, or complex conditions which are life-threatening. Additional characteristics of complex care found in the literature are incorporated (Ward et al., 2015; Carter and Bray, 2017; Brenner, et al., 2018). These are the involvement of multiple agencies, a high burden of daily care, and health prone to rapid change (Whiting, 2012; Woodgate et al., 2015; Pinney, 2017). The cohort definition was developed from synthesis of this evidence base and researcher reflection on clinical practice. This definition created a cohesive cohort for recruitment to the study by identifying similarities in care need and future uncertainty, despite diverse individual diagnoses.

The study sought to address gaps in current research by asking parents to share their experiences of what works well and what needs improvement in terms of care and support provision received (Hartley et al., 2021). A particular focus on decision making and what matters most were chosen because of anecdotal experience highlighting a need for service improvement in these areas and gaps noted in paediatric palliative care literature (Booth et al., 2018). Gathering experiences about current practice generates new evidence and can facilitate local service change (Kings Fund, 2013).

## Aim

To explore the experience of parents caring for a child with complex health in relation to four key areas of enquiry: impact on family life; care/services which work well and areas for improvement; decision making; what matters most.

### Methodology, design, and setting

A qualitative methodology was chosen to enable use of individual participant stories and generate meaning from analysis of their interview responses to produce shared themes (Braun and Clarke, 2013). Semi-structured single telephone interviews maximised flexibility of discussion within a framework, minimising time burdens while creating opportunities for participants to express their views (Polit and Beck, 2013). Participants were recruited from one non-acute community service in Eastern England. The unplanned opportunity to capture qualitative data about experiences during COVID-19 enabled comparison of findings with other emerging evidence (Together for Short Lives, 2020; Gallegos et al., 2022).

### Population

Purposive sampling was achieved by nomination of participants by the Community Children’s Nursing team, which supports a local population of children within the community who have an acute or chronic nursing need. The number of participants was determined by the availability of parents who were eligible and agreed to participate, and generating sufficiently rich interview data to demonstrate patterns through analysis (Braun and Clarke, 2013). Non-English speakers were excluded due to limited access to interpreters at this stage in the pandemic. Fewer participants are needed when exploring sensitive topics because depth within data is generated more quickly (Braun and Clarke, 2013).Participants were included if their child met the cohort definition (Figure 1), were under the care of the Community Children’s Nursing team, and could give informed consent. Participants were excluded if the researcher was their child’s named nurse, if their child’s health was under the safeguarding team, was unstable or deteriorating at that time.

### Data collection

Participants were interviewed individually in September 2020, after the first lockdown of the pandemic in England, using an interview topic guide to maintain focus, sensitively manage emotive issues and to avoid being overly prescriptive (Whiting, 2012). This topic guide was generated from study aims and gaps in existing knowledge (Booth et al., 2018; Hartley et al., 2021). Interviews started with general questions about members of the family and daily care tasks plus experiences of lockdown and shielding, before moving onto discussion of topic areas (Table 1). Telephone interviews increased choice over timing, eliminated physical lone worker risk and met COVID-19 secure research requirements (Braun and Clarke, 2013). However, this method prevented the use of non-verbal cues, so a flexible, collaborative interview style was vital (McLaughlin, 2012). Video consultation facilities were unavailable to participants at this stage in the pandemic.

**Table 1.** Interview topic guide. The example questions are indicative only of the interview style – questions will be open-ended to encourage discussion, with the use of active listening and reflection back to check understanding. The interview will be informal in style to encourage participant ease and honesty. Interview flow may be fluid and topics covered in an order which suits the conversation. Topics overlap so may be covered naturally in the course of the interview. Participants will be reminded that they can choose not to answer any questions as they wish.

	Question examples
**Topic 1**: General experiences of caring for a child with complex health in the home	• What is it like for you at home day-to-day parenting your child with complex health?
	• Can you give me some examples of the care you are doing each day to keep them at home/stable?
	• What kind of support do you have in place at home?
	• Is this helpful?
	Potential prompts: Care packages or carers, parenting issues that are different due to child’s complex health, unexpected issues, information re pandemic
**Topic 2**: Impact on the family	• Have you made any adjustments as a family since child became unwell/health became complex?
	• Where do you get support from?
	• What difference has your child’s health/needs made to the family as a whole?
	• Do you get any ‘me’ time? How?
	Potential prompts: Impacts on siblings, daily activities, work, local support, who can look after child if you’re not there, have you needed any training
**Topic 3**: Decision making	• Thinking about a time when you needed to make a decision about your child’s health care/treatment, what was the process leading to decision/outcome?
	• Can you think of an example when a decision felt easy to reach, do you mind describing that and talking about why it was the right decision?
	• Can you think of a time when a decision was challenging, what was hard? What could have made it easier?
	• Do you feel included in decisions, does your child?
	• Have you been asked to make any emergency plans, what was that like?
	Potential prompts: Family dynamics, equal involvement, helpful support groups/information/resources, social media impact, key professionals who help, what do you do if don’t agree with decisions, emergency plans/advance care planning
**Topic 4**: Care that is good/what could be improved	• What support or care has worked well for you?
	• Thinking about the care your child has received over the last few months, what has been the most helpful?
	• How has it been if you have asked for help or changes to care? How responsive have professionals/teams been?
	• Are there things that haven’t worked well?
	• Do you have any suggestions for improvements to local health care?
	Potential prompts: Specific teams/services/professionals who have been helpful, why, key areas where things haven’t gone well, have changes been made if needed, was that easy/hard to ask for
**Key statement:** What matters most	Final question: If you could say in a nutshell what matters most to you and your child, what would it be?

Participants were not asked to review interview transcripts to minimise time burdens for them. Instead, summary discussion at the end of interview enabled reflection on key areas, meanings and participant satisfaction.

### Ethics

Ethical approval was obtained from the Health Research Authority (Protocol number 19/EE/0265). Written participant information was posted with an invitation to take part, explaining the study in plain English to ensure valid informed consent (Health Research Authority (HRA), 2020). Interested parents had a telephone call with the researcher prior to interview to check understanding and answer questions. Consent was obtained via telephone prior to interview and files stored separately from the data on a secure University server. Demographic details and reference to specific diagnoses were purposely excluded to maintain participant anonymity (Health Research Authority, 2020). Exploration of emotive issues was handled sensitively, and participants could pause or stop interviews at any time (McLaughlin, 2012). Signposting to after-care support was provided.

### Data analysis

Interviews were transcribed verbatim, pseudonymised and analysed (by EW, a novice researcher) using Braun and Clarke’s six phase inductive thematic analysis which ensured rigorous, accurate data capture and processing (Cypress, 2017). Field notes were made immediately after each interview and checked again after coding to minimise personal bias (Braun and Clarke, 2013). Data immersion occurred through transcription of audio recordings via headphones to a typist, followed by repeated listening and re-reading of transcripts. Each interview was coded as a sequence of short statements: these were then collated by searching for patterns across the whole data set (Table 2). Modification and clustering of codes by hand produced potential themes which were reviewed in relation to how they answered the research objectives, as well as the depth of data within each theme across interviews. Quotes were categorised per theme from each interview. Discussions were held at each stage of data analysis between the first and second author (an experienced qualitative researcher). In addition, independent analyses of transcripts were carried out by the second author to ensure trustworthiness of findings: essential for ensuring quality and authenticity of results (Cypress, 2017). Reflexivity requires that qualitative researchers actively reflect on their influence on data generated during a qualitative study (Avis and Reardon, 2008). This was achieved through using a reflective journal to record observations and ideas during and after interviews, and during analysis: increasing self-awareness within interpretation and aiding cross-referencing during supervision.

**Table 2.** Demonstrating the stages of thematic analysis using the Braun and Clarke six phase method, through the example of theme 3.

Step 1: Immersion in data	Step 2: Generating initial codes	Step 3: Searching for themes	Step 4: Review of potential themes	Step 5: Defining and naming themes	Step 6: Reporting
Re-reading transcripts	Searching for patterns across data	Review codes to identify similarity and overlap	Independent analysis	**Theme 3: Being proactive**	Copying and pasting key quotes for each theme into a summary table
				*‘I still don’t agree, and I must look into it a bit further and see what the consultant says’*	
Highlighting each new code/idea with a different colour	Proactive	Proactiveness	Collaboration, discussion, reflection	*‘You have to kind of find it and sometimes guide it yourself for something to happen’*	Building on themes within the report to represent a complete picture
	Juggling	Juggling competing demands	Reviewing miscellaneous codes	*‘You always just want somebody else to be proactive, yeah it is proactiveness really’*	
	Prioritising		Reviewing distribution of potential themes across all transcripts	*‘In the end we said, c’mon let’s get the allen keys and do it ourselves’*	
	Competing demands		Checking potential themes relate to study aims	*‘Us making that decision and getting that second opinion was helpful because, going forward, knowing that is an option’*	
	Conflicting opinions			*‘You’ve got to do things to improve the service. You can’t complain if you haven’t done anything to help’*	
	Holding onto values				

## Findings

Parents (individual mothers, fathers or couples) from 19 families were invited and seven mothers volunteered and were interviewed. Telephone interviews lasted from 45 to 90 minutes. Mothers had children whose ages ranged from 3 to 18 years, were under the care of multiple specialist medical teams, with additional health and social services in both acute and community settings. Three participants’ children received Children’s Continuing Care and all were under Children’s Hospices. Some were single mothers, all had other children, some of whom had additional needs.

Mothers were undertaking a breadth of daily care for their children including: enteral feeding; central line access; administration of medication and/or parental nutrition; seizure management; modified oral diets; multiple medications including trial regimes; suctioning; tracheostomy care; and home ventilation. Some children had undergone innovative treatments in England and abroad; others were waiting for treatment or surgery which was impacted by the complexity of their health. Some children had learning disabilities.

### Themes

Seven distinct themes were identified from the data: cherishing normality; navigating the system; being proactive; meaningful connections; beginner to expert – and back; they’re not any child – they’re my child; and COVID-19. In addition, all mothers made improvement suggestions which were collated as practice recommendations. Quotations are given with pseudonyms.

### Cherishing normality

Mothers described busy lives dominated by care-giving tasks and appointments, all impacting their time, sleep, and leisure. They spoke about unpredictability of their child’s condition affecting family activities:

“You plan something and then you end up in hospital, so we don’t plan, we just do” Beth

Having time at home was crucial and participants spoke positively about being together as a whole family, cherishing this as their new normal. Descriptions included reference to occasions when care facilitated family time, and how this motivated mothers’ decision making.

“Anything that can allow us to be together and do as normal things as possible, like getting Felix up and Freya sitting on his lap on the wheelchair. Going round the block, having Felix sat at the table with the others” Fiona

The impact of hospitalisation on families was also highlighted. When time at home with the whole family was scarce, participants valued connection between services:

“I wish there was more communication with the community team because they get a sense of what we’re dealing with at home” Julie

It was important that care optimised normality and supported the whole family’s needs as well as the child’s. The health and social care system was described as the biggest barrier to quality family time.

### Navigating the system

Mothers expressed gratitude for care and treatment which had enabled their child to survive. However, moments when participants expressed anger or frustration all fell into this theme. Examples included frequent liaison with professionals, chasing up, appointments ‘*like buses*’, protocols which didn’t meet their child’s needs, and repetitive administrative tasks.

“That’s kind of one thing that we struggle with because there’s so many different teams and hospitals and it’s a lot of chasing on our part and liaising between everybody: telling everybody what the other teams have said” Gina

Participants described how time was needed to learn specific cultures of health settings, exacerbated by the number of agencies involved. Examples were given of services having different approaches with no ‘joined-up thinking’ or oversight. Additional challenges were fluctuating health of their child, a need for clear expectations of services, and navigating the personalities of professionals. Barriers such as referral processes and complaint procedures were described, with communication a constant issue: they were learning by experience and with minimal support. Mothers described impacts this had on their energy, time and motivation:

“You feel like you’re banging your head against a brick wall, because you just think what’s the point? Why am I bothering, I might as well just leave it cause I’m not getting anywhere” Anna

There was a common language around recognising when something was important enough to *‘push for’* and when not to try. Descriptions were given of processes which victimise service users and damage coping. Disagreements with professionals were not always negative – participants gave examples of how resolving differences brought long-term benefits without damaging relationships, which they had feared. There was a consensus that having advice and advocacy would improve experiences. Mothers described value in shared knowledge with either peers or professionals and having someone advocating for them.

### Being proactive

All mothers described occasions when they were intentionally proactive, including instances of juggling competing demands, either due to the system or needs of other family members. Instead of being passive recipients, mothers described actively pursuing improvements to the lives of their child and family, or care received:

“*You always just want…to be proactive…You don’t just want to be thinking, ‘well is this it?’ you don’t just want to be settling” Carol*

Being proactive was sometimes driven by a desire for normality, such as balancing siblings’ needs, acquiring health skills to gain time at home and creative problem-solving.

Mothers’ sense of coping in the long-term was directly affected by how proactive they felt – this offset some of the difficulties of living with uncertainty:

“I needed to feel confident that I had come to peace with each of the decisions because I needed to answer to myself and not regret” Debbie

Mothers sought professional input proactively and were keen to benefit other families by sharing examples of ways they had resolved issues. Feedback after interviews showed that participation in this study was another way for them to be proactive.

### Meaningful connections

All participants mentioned the significance of a long-term trusted relationship with a specific professional within health or social care, whose role varied from carer to nurse to social worker to consultant. Mothers described feeling valued and supported because this professional provided continuity and had a family-oriented perspective.

“He uses my name regularly. So you’re still you, and without knowing it, that’s what he’s doing. You’re still you amongst all these other families and situations. ‘I’m looking after David and I’m looking after you’… For those few moments we are the ones that matter” Debbie

Participants articulated how developing relationships with parents in similar situations and a network of friends and family also influenced their resilience. All mothers commented on difficulties maintaining friendships and accessing formal peer support, but that it is beneficial if it occurs, even by chance. Transient and virtual human connections could also have significance.

“It’s like oh *sigh* and you know that could’ve been facilitated earlier, to make you feel that you’re not alone, that you’re not isolated” Carol

### Beginner to expert – and back again

Mothers reflected on their journey as a parent in a medical world, citing skills acquired and challenges they still experience entering new phases of care. Such phases included new treatment, acute deterioration and transitioning between services. Participants highlighted how their own identity was directly impacted by their child’s health status. Mothers described occasions when professionals did not recognise or respect parental expertise, which had a detrimental effect on themselves and their child’s care:

“It’s been horrible…people not understanding her condition and I don’t know whether it’s the fact it’s coming from the parent but people underestimate how much parents understand about their child’s condition” Gina

There was a sense of pride in participant’s knowledge of specialist skills and expertise regarding the unique needs of their child. However, they could rapidly feel like a novice if anything disrupted their status quo:

“Each time a new phase starts, that same feeling of being a beginner again with some overarching things…You gradually gain a little bit of confidence…and suddenly, all of your instincts are knocked off” Debbie

Mothers gave examples of positive interactions with medical and nursing staff, appreciating a team spirit. This was counterbalanced against times when they felt under pressure to make a difficult decision, especially if their child was acutely unwell. Participants expressed a wish to ‘*just be Mum*’ sometimes and pause from care-giving. Barriers to this could be personal (maintaining control or being protective) or professional (staff expressing their own inexperience in relation to the child’s complexity). Mothers’ perceptions of their own expertise gave them confidence at times but could also weaken trusting relationships. A lack of self-confidence at vulnerable times could undermine their ability to participate in decisions.

### They’re not any child – they’re my child

This theme captured descriptions from mothers about their child’s worth, incorporating emotions of pride, distress, and injustice. There was a sense of gratitude for professionals who individualised care, being mindful of their child’s health complexities and their value as a person.

“I think one of the things that matters most is that there is a genuine appreciation of what’s at stake…it’s not a patient…it’s not anyone’s kid, it’s my kid” Debbie

Staff compassion and recognition of their child’s unique attributes were highlighted, with an emphasis on quality of care and mothers were champions of their child’s needs. When discussing decisions they gave examples of situations when they felt their child might have been denied treatment due to their condition or disability:

“His attitude changed, it visibly changed because Jess was disabled…he just assumed Jess was a no-hoper because she was on a sling… thinking ‘why are we bothering’ sort of thing. Well, because she’s my daughter and she’s lovely and she deserves the best” Julie

Mothers were sensitive to others’ perceptions of disability and illness, giving examples of knowledge they purposely acquired to defend their child’s rights: information, written care plans and staff who knew them and could advocate. Public reactions were also referred to, including descriptions of challenges linked with visible and invisible disability.

### COVID-19

Experiences of COVID-19 were influenced by stability of their child’s health during lockdown and whether service changes hindered perceived progression of care. Mothers whose child’s health was less stable or had less established care raised more negative issues. Acceptance of home support whilst shielding was influenced by participants’ sense of coping.

COVID-19 heightened the impression mothers already had of living *‘slightly in your own bubble’*. Participants described difficulties assessing the scale of risk to their child and limited, inconsistent information. Feeling responsible for protection of their child’s health caused added anxiety, with mothers fearful of illness requiring hospital attendance. Receiving letters about resuscitation status heightened their distress:

“I got one of these letters from the GP and it has on it about DNR” Anna

“If you’ve got a disabled person, if it’s a child or what, there were these rumours… somebody else would be more deserving of say life saving treatment” Carol

Unexpected positive effects of lockdown were also emphasised, including increased quality time at home, streamlined care and a deeper cultural awareness of living with uncertainty. Mothers were keen to highlight how positive adaptations could continue into the future, giving practical tips about individualising delivery of health care.

## Discussion

In keeping with the aims, study findings illuminate care and support mechanisms which work well for mothers of children with complex health needs, identify practical service recommendations, and illustrate what matters most within care and decision making. The cohort definition aided recruitment and enabled findings to be compared with existing evidence. Although the participants’ children had a range of complex health needs, all had a large number of teams involved, a high burden of daily home care and unpredictable health. This study provided a positive outlet for mothers by valuing their opinions which directly influenced local service improvements (Kings Fund, 2010).

Findings demonstrate the importance of normality and family time to mothers, and how this can motivate their decisions, supporting existing evidence that families need to feel individually important within a complex system of care, and for children to thrive, not just survive (Coughlan et al., 2020; Mitchell et al., 2022). Findings confirm that rigidity of services and a large number of teams negatively impact a sense of normality and communication (Ward et al., 2015). Participants felt that their children’s needs blurred the boundaries of services, reinforcing the importance of integrated, holistic care (NICE, 2022). This study confirms that optimising communication between families and teams reduces parental anxiety and promotes quality time (Constantinou et al., 2019).

Mothers were clear that respite and support is essential but under-promoted and parental coping mechanisms can delay acceptance of help. Virtual and physical support networks lessened feelings of isolation and promoted sharing of experiences, which helped to protect their identity and sense of purpose. It is possible that this study appealed to proactive mothers who had learned to prioritise and persevere. Findings highlighted this as a positive mechanism for mothers, which deepens evidence on assertiveness and battlegrounds (Swallow et al., 2011; Whiting, 2012).

This study’s findings provide new evidence that a sense of expertise can be transitory and context dependent. It is important to recognise mothers’ levels of expertise in relation to their child without undermining their primary need to be ‘Mum’ (Carter and Bray, 2017). Findings confirm that decision making is a challenge at times of increased vulnerability, such as acute deterioration of their child’s health (Durrall et al., 2012). Three values were highlighted during interviews: trust, hope and truth. Mothers spoke about hope enabling them to face each day balanced with perceived truth of their child’s condition. Weighing these up has been found to impact parental decisions (Hinton and Kirk, 2016; Mitchell et al., 2019). Opportunities to revisit difficult choices and challenge decisions (without formally complaining) was beneficial, adding further evidence that exploring options can reduce parental experiences of regret (Zaal-Schuller et al., 2016).

Findings show that it is beneficial to acknowledge barriers to quality parenting time and increase support at times when confidence dips: during new diagnoses, periods of health instability and transition. Effective care and communication regardless of a child’s perceived complexity is vital for parent and child. In keeping with Mendes’ (2013) findings, participants in this study confirmed that key areas of care were kindness, listening, taking an interest, and ensuring access to comfort and safety measures. Facilitating sharing of experiences was recommended by mothers for themselves, other families and service improvement.

The study was planned prior to the outbreak of the pandemic and gathering experiences directly relating to COVID-19 was not an original aim. However, because the study was carried out during the pandemic, participants readily shared their experiences and their inclusion was an unexpected addition to the study. Mothers expressed heightened anxiety during COVID-19 in relation to potential availability of treatment for their child, commenting on difficulty ascertaining reliable advice. This may provide a rationale for data showing delayed acute presentation to hospital and reduced admissions during lockdown for this patient group (Markham et al., 2022). These qualitative findings add to emerging evidence that parents of children with complex needs have experienced increased burdens of care during COVID-19 and felt socially isolated and invisible (Mathilde, 2021). Findings concur with evidence that parental experiences were more negative for those whose children’s health was unstable or lacked established professional support (Gallegos et al., 2022). This study demonstrated positive and negative impacts of COVID-19 which are supported in the literature: lack of care progression was sometimes mitigated by new strategies such as flexible access to appointments, contingency planning, and interagency collaboration (Jones et al., 2020; Markham et al., 2022).

### Limitations

Due to participant uptake this study did not capture the voice of fathers, whose role is important and under-recognised (Fisher et al., 2021). The study included a small, narrow participant demographic, excluding non-English speakers and parents from other sites, limiting the generalisability of findings (Polit and Beck, 2013). However, valuable and reliable evidence can be provided by small studies and related to a field of practice (Marjanovic et al., 2019). It is hoped that by highlighting the complex care experiences of the mothers who are the focus of this study, the findings may provide insights for both acute and community settings.

## Implications for practice

**Figure 2.**
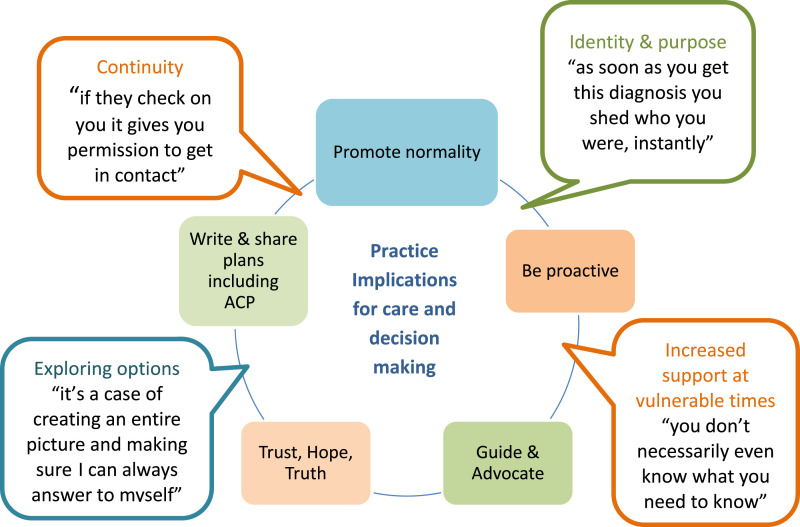
Implications for practice.

Inclusion of practice implications was embedded in the study aims to ensure exploration of interventions which help this group of parent-carers (Hartley et al., 2021). During interview mothers highlighted specific areas which would enhance care and support from professionals for their children with complex health, summarised in Figure 2 as:

• Promoting normality and being proactive
• Guidance and advocacy
• Coping and a sense of purpose
• Trust, hope, and truth
• Early Advance Care Planning

Findings highlight that regular informal check-ins with families can provide continuity and compassion, without making them feel checked-up on (Mendes, 2013). Providing empowering support requires flexibility and sensitive understanding of a family’s current situation. Mothers valued direct access to key professionals, promotion of time at home and achieving ‘normal’ childhood milestones. Service adaptions arising due to COVID-19 which have created flexible access to professionals and virtual methods of connection and community between families should be continued (Jones et al., 2020).

Providing guidance, advocacy and allowing meaningful relationships to flourish helped mothers feel less alone. Findings confirmed this applies to interactions both with professionals and peers (Smith et al., 2013). Participants wanted to be empowered with skills and knowledge to help them manage and connect with others. Availability of support to build resilience must be tailored to individual need, not limited by time constraints, finances, or physical access. Selective use of specialist charity networks and social media support benefitted mothers; they were keen for guidance in identifying reliable sources of information and support.

Building trust through daily decisions has been shown to result in more effective communication when facing difficult choices (Mitchell et al., 2019). Discussing family goals and values and clarifying what matters most was helpful within interviews and is also recommended in clinical practice (Picton-Howell, 2020). Some mothers described positive outcomes through resolving disagreements with professionals: increased confidence, honesty, and compassion. Evidence of conflicts in children’s healthcare supports this and emphasises the importance of recognising parental expertise (Parsons and Darlington, 2021).

Introducing Advance Care Planning (ACP) conversations during parallel planning – establishing goals for life as well as potential deterioration – was considered appropriate and beneficial (Beecham et al., 2016; DeCourcey et al., 2018). Mothers verified that such conversations are difficult but necessary, and permission-seeking and a family focus can help (Sidgwick et al., 2019; Orkin et al., 2020). These conversations are known to reduce anxiety and benefit children for whom full resuscitation and keeping options open is appropriate (Allen, 2014; Horridge, 2015). A key element affecting ACP for mothers was experiences of actual and perceived discrimination in relation to disability and health complexity. Research has previously demonstrated that this can occur: it is essential that professionals do not assume that because a child has an ACP they are not for active treatment (Picton-Howell, 2020). This study found that when written plans were made, mothers felt reassured about access to appropriate emergency care for their child.

### Future research

Further evidence on children’s complex health should include experiences of young people, whose perspectives remain under-represented (Booth et al., 2018). Evaluation of specific targeted support for parent-carers, both formal and informal, would help to drive and focus funding for service changes. Research which reviews the use of specific decision making tools would be of practical benefit and promote family collaboration throughout the patient journey (Joseph-Williams et al., 2014).

## Conclusion

Improving maternal support is essential to promote their health and resilience whilst caring for their children. Participants cherished family time at home with their child, which influenced decision making. They were intentionally proactive to ensure optimum care and navigate the health and social care system. Advocacy and guidance particularly help when mothers doubt their expertise or their child’s health is unstable. Involvement in decisions can be difficult but is important: permission-seeking, discussion of goals, and holistic appreciation of the family are beneficial. Early conversations and written ACP can ensure access to appropriate treatment regardless of disability or complexity, whilst reducing anxiety. Allowing mothers to share their stories enhances their wellbeing and can foster a sense of purpose.

New qualitative evidence on the impact of COVID-19 highlights the relationships between maternal experience and the stability of their child’s health, their sense of expertise and availability of supportive connections. The on-going struggles which families have with fragmented care and isolation have been exacerbated, whilst opportunities have arisen to adapt services according to individual need.
